# To what extent are patients involved in researching safety in acute mental healthcare?

**DOI:** 10.1186/s40900-022-00337-x

**Published:** 2022-02-28

**Authors:** Lyn Brierley-Jones, Lauren Ramsey, Krysia Canvin, Sarah Kendal, John Baker

**Affiliations:** 1 School of Healthcare, University of Leeds, Leeds, UK; 2 Yorkshire Quality and Safety Research Group, Bradford Institute for Health Research, Bradford, UK; 3 Leeds Institute of Health Sciences, Leeds, UK

**Keywords:** Interventions, Inpatient, Mental health, Patient involvement, Safety, Research

## Abstract

**Background:**

There is a growing need to involve patients in the development of patient safety interventions. Mental health services, despite their strong history of patient involvement, have been slow to develop patient safety interventions, particularly in inpatient settings.

**Methods:**

A systematic search was undertaken of both academic and grey literature. Whilst no lay member of the team worked directly on the review, they were part of the project steering group which provided oversight throughout the review process. This included people with lived experience of mental health services. From a research perspective the main focus for lay members was in co-producing the digital technology, the key project output. Smits et al.’s (Res Involv Engagem 6:1–30, 2020) Involvement Matrix was used to taxonomise levels of patient involvement. Studies were included if they were set in any inpatient mental health care context regardless of design. The quality of all selected studies was appraised using Mixed Methods Appraisal Methodology (MMAT).

**Results:**

Fifty-two studies were classified, synthesised and their levels of patient involvement in the research and development of patient safety interventions were taxonomised. Almost two-thirds of studies (n = 33) researched reducing restrictive practices. Only four studies reported engaging patients in the research process as decision-makers, with the remaining studies divided almost equally between engaging patients in the research process as partners, advisors and co-thinkers. Just under half of all studies engaged patients in just one stage of the research process.

**Conclusion:**

Involvement of patients in researching patient safety and developing interventions in an inpatient mental health context seems diverse in its nature. Researchers need to both more fully consider and better describe their approaches to involving patients in safety research in inpatient mental health. Doing so will likely lead to the development of higher quality safety interventions.

## Abstract (plain language summary)

We know that inpatient mental health settings are not safe. By getting patients to help us research and develop interventions to improve safety they are more likely to work. We searched for articles and papers which described doing this. By using a matrix we were able to understand how many research studies involved patients. We found 52 studies, but few really involved patients in the entire research and intervention development process. Most of the research focus was on helping staff to use less restrictive interventions like restraint and seclusion. Only four studies really treated patients as equals in terms of decision making in the research process, and about half of the studies involved patients in only one of the possible three stages of research. There have been lots of ways patients have been asked to be involved in research but we really need to improve the way we involve patients in order for our knowledge about patient safety and the interventions that follow from this to be truly co-produced.

## Background

Patient safety is a global health priority. The World Health Organisation (WHO) defines patient safety as a discipline that aims for ‘the absence of preventable harm to a patient during the process of health care and reduction of risk of unnecessary harm associated with health care to an acceptable minimum’ [1, 2] and for the improvement of safety of healthcare broadly [3–5]. The term ‘patient safety’ can, however, mean different things to staff and patients [8], as well as between patients [6], and patients have been shown to engage with some safety related behaviours and not others [7].  Lawton et al. [9] identify three types of patient involvement in safety research and interventions that may be found in different areas of safety; patients intervening directly/patient-mediated approaches (e.g. by reminding staff to wash their hands), patient education to be better able to manage their treatment (e.g. self- management of medicines) and patient feedback on care safety (e.g. discharge or inpatient surveys). Patients have a clear role in improving the safety of healthcare broadly and their involvement in this area of research is fundamentally important [10].

Recent UK evidence suggests that patient safety in acute mental health care is particularly challenged. A Care Quality Commission (CQC) report [11] revealed that 36% of National Health Service and 34% of mental health independent core services, which includes inpatient care, required improvement in the safety domain. The CQC also reported an over-use of detention in mental health services under the Mental Health Act (2007), leading to a risk averse, rather than therapeutic, culture [12]. More recently, an independent review of the Mental Health Act of 2018 expressed concern over “the disproportionate number of people from black and minority ethnicities detained under the [Mental Health] Act” [12].

In 2020, the National Reporting and Learning System (NRLS) reported a total of 204,307 safety incidents between 1st October 2019 and 31st March 2020 from all Mental Health Trusts in England [13]. Of these, only 770 (0.4%) were classified as severe, 11,520 (6%) as moderately severe, 67,130 (33%) as of low severity and 123,674 (61%) as causing no harm. The most frequently reported incidents concerned self–harm (n = 48,195 24%), were care/monitoring errors (n = 34,049 17%), and concerned disruptive, aggressive behaviour (n = 22,456 11%). A total of 1,213 deaths from errors were reported (0.6%).^1^

Given the lack of safety in acute mental health care there is a clear need for improvement but research in this area has reportedly lagged that in other areas of medicine [14, 15] despite a long user movement history. Moreover, patients and providers reportedly disagree about which outcomes matter most in mental health safety [16, 17]. Where patient involvement has been incorporated into safety research in inpatient mental health, service evaluations have been shown to be more user friendly and the outcome measures more relevant [18]. Nevertheless, patient involvement in safety research in inpatient mental health care presents challenges; patients with mental illness may lack mental capacity, and detention under the Mental Health Act (2007) may result in diminished freedoms [19] leading to lower levels of social participation and hindering contributions [20]. Moreover, patients’ symptoms and treatment regimens may limit their willingness or capacity to engage with research [19]. Patient involvement in research may be hindered by negative patient experiences and by limited and tokenistic support for their involvement [20]. Some contend that perhaps, because of these challenges, acute mental health care has been slower to adopt the co-production techniques of general health services in the context of patient safety [9].

This review aimed to identify those studies which involved patients in research which was designed to improve patient safety in the acute mental health care context. Using the Involvement Matrix of Smits et al. [21] we classified reported patient involvement in relation to the design, delivery, implementation and/or evaluation of research studies and interventions. Within these patient roles in research we also assessed levels of patient involvement; that is, from passive to active, the former referring to patients simply receiving, or being asked for, information and the latter to patients being active decision-makers.

## Methods

### Inclusion and exclusion criteria

Studies were included if they involved patients in researching  and improving the safety of patients and/or staff. Studies were excluded if the study (a) was not safety focused (b) focused only on medication error/contraindication (c) reported low involvement of patients, that is, as listeners or as research participants only, according to Smits et al.’s Involvement Matrix [21] (see Appendix 1) (d) focused only on patient complaints or feedback (e) focused on mental health safety but did not include an intervention, and (f) focused on misdiagnosis through language barriers. With these exclusion criteria in mind we took the term intervention to mean any product, activity or process that aimed to reduce the risk of harm and/or increase safety in acute mental health.

### Outcomes

Outcomes related to patient involvement in the design and/or delivery and/or implementation and/or evaluation of any patient safety research and intervention in acute mental health care.

### Settings

Studies were included if they were set in any inpatient mental health care context including Psychiatric Intensive Care Units (PICUs) and forensics. Studies were excluded if they were set in care homes, prisons, primary care, community care settings, general emergency departments, or related to secondary care discharge and post discharge (unless patients were moving to alternate secondary/tertiary care settings) and schools.

### Study designs

All study designs were included if they reported patient involvement in safety research and interventions, including quantitative and/or qualitative methodology, Quality Improvement (QI) studies and those using Plan, Do, Study, Act (PDSA) cycles.

### Search strategy and study selection

Database and grey literature sources were systematically searched using key words related to ‘mental health’ and ‘patient safety’, Medical Subject Headings (MeSH) related to pre-specified Population, Intervention, Comparison, Outcomes, Study Design (PICOS) criteria and the research question: ‘To what extent are patients involved in interventions to improve patient safety in acute mental health care?’ Six databases (CINAHL, PsycInfo, Medline, Embase, Web of Science and Scopus) were searched (terms listed in Appendix 2) and results were collated in EndNote to remove duplicates. Review papers were not excluded from the search but none produced by the search fit our inclusion criteria exactly. Review papers produced by the search were scanned for potentially relevant papers and any found were extracted and evaluated as individual papers.

The inclusion of grey literature in the search was considered beneficial because much research and innovation is conducted by clinical teams in the area of safety in acute mental health, but findings are often not reported in the published literature. Twenty-five non-mental health specific sources (e.g. Royal College of Nursing) and 14 mental health specific sources (e.g. Centre for Global Mental Health) were also explored. Non-mental health specific resources were identified via expertise within the research team and through previously explored sources from similar recent projects. Two relevant databases (National Institute for Health and Care Excellence (NICE) Evidence (https://www.evidence.nhs.uk/) and ProQuest Thesis and Dissertations) and three social media platforms (Twitter, Facebook, YouTube) were also searched. Where available, the first 100 returns from each source were screened according to the eligibility criteria. Additionally, known grey literature sources of interest based on authors’ expertise were included via hand searching and screened according to the eligibility criteria. A database search produced 13,923 unique citations.  Search results were exported to Covidence for screening at title, abstract and full text level by two reviewers (LBJ and SK). Disagreement on the selection of studies was discussed until consensus was reached. The Preferred Reporting Items for Systematic Reviews and Meta-Analyses (PRISMA) flowchart is presented in Appendix 3 [22]. Title and abstract screening left 272 papers for full-text review. After full-text screening, a further 249 papers were excluded leaving a total of 23 studies from the published literature search. In addition, 12 studies were produced through hand searching and 17 studies/reports retrieved from the grey literature search leaving a total of 52 studies for inclusion in this review. Only studies published after 2000 were included.

### Data extraction and quality assessment

A total of 17,492 studies were extracted using the Covidence online tool and an adaptation of categories using generic features informed by the Joanna Briggs Institute Reviewers Manual [23]. Extracted data included country of origin, study design, duration and quality, setting, sample size and characteristics, principal focus of study, theoretical framework or model, nature and outcome of intervention and level of patient involvement. The quality of all selected studies was appraised using Mixed Methods Appraisal Methodology (MMAT) [24] a tool suitable for assessing studies of heterogeneous methodologies. Studies were classified accordingly as; Quantitative Randomised Control Trial, Quantitative Descriptive, Quantitative Non-Randomised, Qualitative or Mixed Methods studies and were assessed according to the criteria associated with each methodological category. No studies were rejected based on quality.

### Categorising patient involvement

Likewise, reported patient involvement in each study was evaluated. Several extant systems of classification and assessment exist, including Boote et al. [25] who classify patient involvement in terms of consultation, collaboration and consumer control, Greenhalgh [26] who identify  65 frameworks which vary according to their power, priority setting, study or partnership focus, Rose [27] who note the difficulty in levelling power relations between researchers, clinicians and patients and Beresford [28] who conclude that recruitment, funding, parity and the lack of research careers for service users remain ongoing problems in collaborative research [25–28]. Most recently Smits et al.’s [21] Involvement Matrix has been devised. This identifies five patient roles (listener, co-thinker, advisor, partner, decision-maker) and three involvement stages (preparation, execution and implementation). The Involvement Matrix has been designed as both a prospective and retrospective tool and was selected for use in this review due to its breadth of classification, its relevance to the research question and its retrospective applicability. Each study was classified according to the most active patient role reported, and thus assigned to one role only (even though patients may have, for example, been ‘listeners’ as part of their role as ‘advisors’). Each study was classified according to patient involvement in one, two or all three stages of safety research and intervention development, as reported by authors (Table 1). The role of listener was considered to indicate ‘low involvement’ as it engages the patient only as a recipient of information. The roles of partner and decision-maker on the other hand were considered to indicate ‘high involvement’ as they engage patients more fully (such as partnering in designing and delivering safety training) or making decisions (such as sitting on a project management board). The roles of advisor and co-thinker were considered to indicate ‘medium involvement’. Similarly, patient involvement was considered to be ‘high’ if patients were engaged in all three stages of an intervention, namely, preparation, execution and implementation, ‘medium involvement’ if engaged in only two stages (regardless of reported role involvement) and ‘low involvement’ if engaged in only one stage. Unlike roles, the three stages were considered, individually. to offer equal levels of patient involvement.

**Table 1:** Distribution of Patient Involvement

Authors	Focus	Intervention Type	Involvement matrix							
			Roles					Stages		
			Listener	Co-thinker	Advisor	Partner	Decision-maker	Preparation	Execution	Implementation
Maguire et al. [43]	Restrictive practices	6 Core Strategies					X	X	X	X
Qurashi et al. [37]	Restrictive practices	Patient input to clinical practice/staff training					X	X	X	X
Avon and Wiltshire Mental Health Partnership NHS Trust [77]	Restrictive practices	Safewards				X		X	X	X
Lombardo [66]	Restrictive practices	Framework development				X		X	X	X
Care Quality Commission [73]	Restrictive practices	Patient input to clinical practice/staff training				X		X	X	X
Ashcraft et al. [50]	Restrictive practices	No Force First				X		X	X	X
Melvin et al. [44]	Self-harm	Phone app				X		X	X	X
Riley et al. [38]	Restrictive practices	No Force First				X		X	X	X
Riahi et al. [57]	Restrictive practices	6 Core Strategies				X		X	X	X
Fluttert et al. [64]	Violence to others	Tool—Early Recognition Method				X		X	X	X
Scottish Patient Safety Programme [65]	Restrictive practices	Debriefing			X			X	X	X
Bruyneel et al. [63]	General safety	Delphi rounds					X	X	X	
Ashcraft et al. [49]	Restrictive practices	Patient input to clinical practice/staff training					X		X	X
Pfeiffer et al. [48]	Self-harm	Peer support				X			X	X
NHS England [67]	Restrictive practices	Tool—My Safety Plan				X		X	X	
Hampshire Partnership NHS Foundation Trust [75]	Restrictive practices	Tool—Restrain Yourself				X		X	X	
Bowers et al. [31]	Restrictive practices	Safewards			X			X	X	
Loveridge [52]	Self-harm	Tool- Safe-kit			X				X	X
SAMSHA [55]	Restrictive practices	6 Core Strategies			X				X	X
McLellan [78]	Restrictive practices	Tool—Patient safety climate			X			X	X	
American Psychiatric Association [81]	Restrictive practices	Tool—Timetable/co-production			X			X	X	
Riemer & Corwith [79]	Restrictive practices	6 Core Strategies			X			X	X	
Smith & Millar [70]	Restrictive practices	Sensory modulation			X			X	X	
Merseycare NHS Trust [72]	General safety	Patient feedback on Quality Improvement initiatives			X			X	X	
Huckshorn et al. [80]	Restrictive practices	Patient input to clinical practice/staff training			X			X	X	
Lenagh-Glue et al. [61]	General safety	Tool—Advanced Preferences Instrument				X		X		
Le Francois [34]	Emotional/psychological safety	Staff facilitation				X		X		
Price et al. [36]	Restrictive practices	Safewards				X				X
Stensgaard et al. [62]	Restrictive practices	Safewards				X				X
Dipankui et al. [58]	Restrictive practices	Health Technology Assessment			X				X	
Kontio et al. [59]	Restrictive practices	Patient input to clinical practice/staff training			X			X		
Short et al. [46]	Restrictive practices	6 Core Strategies			X			X		
Barrera et al. [30]	General safety	Artificial Intelligence—remote nursing observations			X					X
Brown et al. [32]	Violence to others	Sensory modulation			X					X
Taxis [53]	Restrictive practices	Debriefing			X			X		
Kennedy et al. [41]	Restrictive practices	Safewards			X			X		
Wilson et al. [47]	Restrictive practices	6 Core Strategies		X				X	X	X
South London and Maudsley NHS Foundation Trust [69]	Violence to others	4 Steps to Safety		X				X	X	X
Jonikas et al. [51]	Restrictive practices	Patient input to clinical practice/staff training		X				X		X
Vincent et al. [68]	General safety	Ward rounds/meetings		X				X	X	
Goulet et al. [56]	Restrictive practices	Debriefing		X				X	X	
Curtis et al. [33]	General safety	Ward/building design		X				X		
Horsfall & Cleary [39]	Restrictive practices	Tool- leaflet		X				X		
Hyde et al. [40]	Restrictive practices	Framework development		X				X		
Lantta et al. [60]	Violence to others	Tool-DASA		X					X	
Page et al. [35]	Sexual safety	Qualitative workshops		X				X		
Abou-Sinna & Luebbers [45]	Emotional/psychological safety	Tool- risk assessment predictor		X				X		
Lloyd et al. [42]	Restrictive practices	Sensory modulation		X						X
Wale et al. [54]	Restrictive practices	Sensory modulation		X						X
Appleby et al. [71]	Self-harm	Risk predictor		X				X		
Quinliven et al. (undated) [74]	Self-harm	Input to suicide prevention strategy		X				X		
The Health Foundation [76]	Emotional/psychological safety	Ward rounds		X				X		

### Data analysis and synthesis

Due to the wide range of methodological designs, intervention strategies, nature of patient involvement and areas of mental health safety focus a narrative synthesis method was used [29]. A narrative synthesis approach is particularly suited to analysing the characteristics of, and relationships within and between, heterogeneous studies and, in this instance, in their relationship to the nature of patient involvement, intervention type and outcomes. A narrative synthesis approach as used here was informed by Popay et al. [29]. Textual description, as well as a range of classification categories, formed the basis of a preliminary synthesis designed to reveal patterns across studies.

## Results

### Characteristics of included studies

Fifty-two studies were synthesised in this review which spanned nine national contexts. Nine studies were conducted in the United Kingdom (UK) [30–38] and Australia [39–47], eight in the United States (US) [48–55], three in Canada [56–58], two in Finland [59, 60] and one each in New Zealand [61], Denmark [62], Belgium [63] and the Netherlands [64]. By contrast the grey literature spanned only two national contexts, the UK and US. Fourteen sources were from the UK [65–78] and three from the US [79–81].

### Study design of included research

All 52 studies were classified using the MMAT as follows: two Quantitative Randomised [31, 48] eight Quantitative Non-randomised [36, 42, 45, 48, 50, 55, 64, 74, 79], fourteen Quantitative Descriptive [37, 43, 49, 51–54, 57, 61, 62, 70, 72, 73, 76], nine Qualitative [33–35, 39, 41, 56, 58, 59, 66], and five Mixed Methods [30, 44, 60, 63, 71]. Fourteen studies (all grey literature) could not be classified using MMAT as they used a selection of quality improvement designs including four which used PDSA cycles [38, 40, 65, 69], seven used other QI methods [32, 46, 47, 67, 68, 77, 81] and in three studies the methodology was unclear [76, 78, 80].

### Focus of included research

Studies were classified according to their principal focus. Almost two-thirds of studies (n = 33 focused on reducing restrictive practices [31, 34, 37–43, 46, 47, 49–51, 54–57, 59, 62, 65–67, 70, 77–79, 81]-[53, 58, 73, 75, 80]. Of these, 22 were from the published literature [31, 36–43, 46, 47, 49–51, 53–59, 62] and 11 were from the grey literature [66, 67, 70, 73, 75, 77–81]. Most (n = 22) were evaluated as involving patients at a medium level of involvement [31, 36, 38, 41, 46, 47, 49–51, 53, 55, 57, 59, 62, 65, 67, 70, 75, 78–81]. Five reported engaging patients at a high level of involvement [37, 43, 66, 73, 77] and six at a low level [39, 40, 42, 54, 56, 58]. Studies focusing on restrictive practices with high and low levels of patient involvement are discussed in more detail in Sects. 3.2.3.4 and 3.2.4.2 below (respectively).

Of those studies focusing on restrictive practices, reference was made to eight different interventions: two studies used ‘No Force First’ (NFF) [38, 50]; five used ‘Safewards’ [31, 36, 41, 62, 77]; five used ‘6 core strategies’ [43, 47, 55, 57, 79]; three used debriefing [53, 56, 65]; three used framework development [40, 46, 66]; five used a patient safety/involvement tool [39, 67, 75, 78, 81]); three used sensory modulation [42, 54, 70] and seven used patient input into clinical practice/staff training [37, 49, 51, 58, 59, 73, 80]. Of the remaining 19 studies, six focused on general safety [30, 33, 61, 63, 68, 72], five on self-harm/suicide [44, 48, 52, 71, 74], four on violence toward others [32, 60, 64, 69], three on emotional/psychological safety [34, 45, 76], and one on sexual safety [35].

### Range of safety interventions in included research studies

Safety interventions ranged from macro level interventions (culture/system change at the organisational/policy level) through meso level interventions (decision making and debriefing frameworks at the ward/team level) to micro level interventions (a safe kit/mobile phone app at the individual level). Across all 52 studies the interventions used were; three frameworks (a decision making [40] and QI framework [46]and the PROactive Management of Integrated Services and Environments (PROMISE) governance framework [66]), eight tools (a risk assessment tool [45], a safe kit [52], a patient post seclusion leaflet [39], the Dynamic Appraisal of Situational Aggression (DASA) tool [60], an advanced preferences tool [61], the Early Recognition Method (ERM)[64], a patient safety climate tool [78] and a self -management tool [67]). Five used ‘6 core strategies’ [43, 55, 57, 75, 79], five used ‘Safewards’ [31, 36, 41, 62, 77], four culture change interventions [47, 49, 54, 76], four used patient collaboration/advocacy [37, 53, 80, 81], three sensory modulation [32, 42, 70], three used a technology (health technology to assess alternatives to seclusion and restraint) [58], a phone app to reduce suicide and suicide ideation [44] and Artificial Intelligence (AI) to conduct remote nursing observations [30]), two used ‘No Force First’ [38, 50], two used debriefing [56, 65], two used weekly ward meetings [68, 73] and the remainder (n = 11) were miscellaneous as follows: building/ward design [33], qualitative workshops [35], peer support [48], patient feedback on QI initiatives [72], facilitating children’s voices [34], patient suggested alternatives to seclusion and restraint [59], staff training [51], risk assessment/prediction [71], inpatient input into the national suicide prevention strategy [74], patient input to safety agenda via Delphi rounds [63] and '4 steps to safety' [69]. The type of intervention and focus of each study is summarised in Table 1.

## Patient involvement

### Patient involvement principally in ‘co-thinker’, ‘advisor’ and ‘partner’ roles

**Fig. 1:**
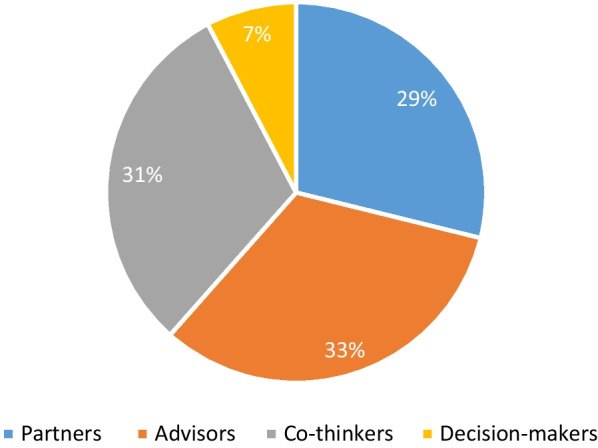
Patient Roles in Selected Studies

Almost a third of studies reported engaging patients in the role of partners (n = 15) [34, 36, 38, 44, 48, 49, 57, 61, 62, 64, 66, 67, 73, 75, 77], a third as advisors (n = 17) [30–32, 41, 46, 52, 53, 55, 58, 59, 65, 70, 72, 78–81] and almost a third as co-thinkers (n = 16) [33, 35, 39, 40, 42, 45, 47, 51, 54, 56, 60, 68, 69, 71, 74, 76]. Only four studies reported engaging patients as decision-makers [37, 43, 50, 63] (see Fig. 1). Table 1 summarises the distribution of involvement across all studies. Studies reporting patient involvement in the least engaged role of listener were screened out at the full text stage. All studies engaging patients as decision makers were from the published literature.

### More active patient roles associated with more extensive patient involvement

Just under half of all studies (n = 22) reported engaging patients in just one stage of safety research and  interventions [30, 32–36, 39–42, 45, 46, 53, 54, 58–62, 71, 74, 76]. Of these, 14 engaged patients in the preparation stage [33–35, 39–41, 45, 46, 53, 59, 61, 71, 74, 76], two in the execution stage [58, 60] and six in the implementation stage [30, 32, 36, 42, 54, 62]. The most common patient role in one stage studies was that of co-thinker [33, 35, 39, 40, 42, 45, 54, 60, 71, 74, 76].

**Fig. 2:**
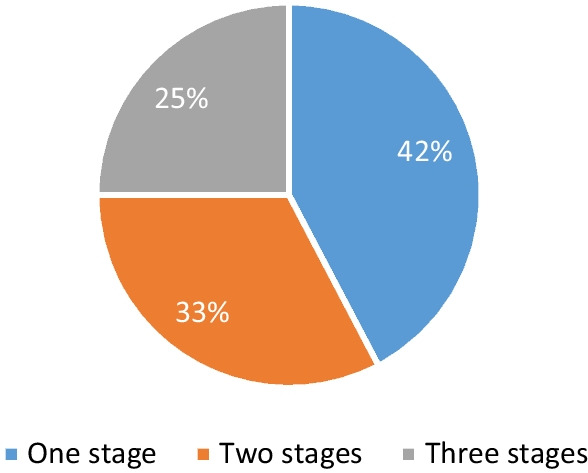
Proportion of Patient Involvement in Selected Studies by Research Stage

One third of studies (n = 17) reported engaging patients in two stages [31, 48, 50–52, 55, 56, 63, 67, 68, 70, 72, 75, 78–81](see Fig. 2) The most frequent two stage combination of  patient involvement was preparation and execution, which represented just under a quarter (n = 12) of all ‘2 stage’ studies [31, 56, 63, 67, 68, 70, 72, 75, 78–81], followed by execution and implementation (n = 3) [48, 50, 52, 55], then by preparation and implementation (n = 1) [51]. Just over half (n = 9) of all ‘2 stage’ studies reported patient involvement in the role of advisor [31, 52, 55, 70, 72, 78–81].

**Fig. 3:**
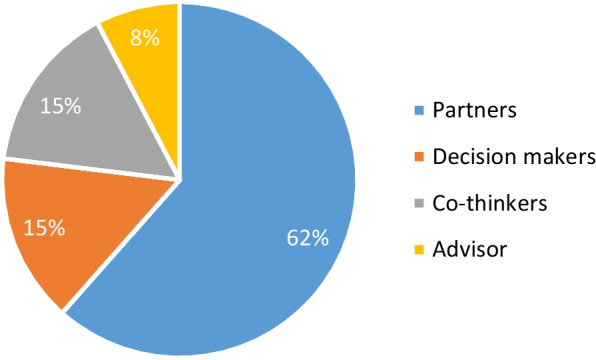
Chart Showing Research Roles of Patients When Involved in Three Stages of Research

Only a quarter (n = 13) of studies demonstrated involvement in all three stages [37, 38, 43, 44, 47, 49, 57, 64–66, 69, 73, 77] (see Fig. 2). Of these 13 studies most were as partners (n = 8) [38, 44, 49, 57, 64, 66, 73, 77], two engaged patients as decision-makers [37, 43], two as co-thinkers [47, 69], one as an advisor [65], and no patients were involved as listeners (as noted earlier these were screened out). Over three quarters (n = 10) of ‘3 stage’ studies reported patients in the roles of partner or decision-maker [37, 38, 43, 44, 49, 57, 64, 66, 73, 77] (see Fig. 3).

From the above it can be seen that associations exist between patient involvement in a single stage of research and the role of co-thinker, patient involvement in two stage studies and the role of advisor and patient involvement in three stage studies and the more active research roles of partner and decision-maker. Thus, more extensive patient involvement and more active research roles for patients frequently occurred together.

### Research with high patient involvement focused on forensic mental health

Only two studies were evaluated as having the highest levels of patient involvement in research and intervention implementation. This was because both studies reported involving patients in decision-making processes and across all three stages of research. Both studies focused on the reduction of restrictive practices in the forensic context, were from the published literature and spanned a 5-year period [37, 43].

Qurashi et al. [37] a UK based study, found that seclusions could be reduced by using advocacy provision, patients’ forums, collaboration between clinicians and patients and patient representatives on ward clinical business meetings, the use of Advance Directives, the building of therapeutic alliance between patients and staff and the process of debriefing. Patients’ views were incorporated into the design and planning of the ward environment, in the development of seclusion policy (key to the research team’s evaluation as high involvement) and in therapeutic risk taking. These interventions achieved over a 60% reduction in seclusion episodes, which fell from 54 to 18 hours per month, with no increase in adverse events over the study’s 5-year period.

Maguire et al. [43] conducted in an Australian setting, implemented the ‘6 core strategies’ (as developed in the US^2^) across five mental health units totalling 116 beds. The authors describe a process of ‘genuine consumer involvement’ which included a consumer consultant being a member of the Project Management group (key to authors’ evaluation as high involvement) and several consumer consultants liaising with representatives of the Consumer Advisory Group (CAG) in order to collate personal experiences of seclusion. Community meetings were used to discuss the research project and resultant initiatives. Patients inspected the seclusion suites and were able to suggest refurbishment ideas, whilst consumers delivered some staff training and consumer consultants, as well as the CAG, were involved in developing the Safety Plan. Staff and patients collaborated to review the unwritten and ‘arbitrary’ unit rules, which were often a source of conflict, and seclusion (and release) processes were made transparent for patients. Admission procedures were also revised, part of which included the implementation of safety plans, a collaborative document completed by the patient with staff that recorded stressors, triggers, warning signs, calming strategies and communication and de-escalation strategies. Patients also took part in post-seclusion de-briefings to enable them to process the experience of seclusion. Maguire et al. [43] succeeded in reducing the frequency of seclusion events by 80% from 100 per month in January 2007 to 20 per month in July 2010 but also the duration by 96% from 5000 seclusion hours in January 2006 to 200 seclusion hours in July 2010. There was less reduction (22%) in the number of patients secluded falling from a high of 18 patients per month in January 2006 to 14 per month in 2010.

Qurashi et al. [37] concluded that a reduction in seclusion incidence is possible when this objective is both a managerial and clinical priority and supplemented by robust performance monitoring and effective clinical governance arrangements. Maguire et al. [43] cited the main challenges to seclusion reduction as being patient characteristics, prisoner culture and the need to ensure safety. Maguire et al. [43] concluded that staff awareness was heightened during the study and this combined with the reduction initiatives to reduce the frequency and duration of seclusion episodes. The enhanced practice of staff may have been sufficient to reduce ongoing aggression but was insufficient to prevent it in the first instance. The authors conclude that the previous complacency of staff, who had potentially used seclusion for behaviours presenting no immediate danger (e.g. verbal abuse), was replaced by a view of seclusion as therapeutic.

Methodologically, Qurashi et al. [37] conducted a good quality study using quantitative descriptive data in a retrospective analysis. As the authors acknowledge, the generalisability of findings may be limited due to its intervention population consisting entirely of male patients whose main morbidity was schizophrenia and its sub types. Nevertheless, all five MMAT methods criteria are reported (where relevant). Maguire [43] also conducted a study generally of good methodological quality, although the results are poorly presented. It benefits from greater representativeness than Qurashi et al. [37], in that both male and female patients were involved in the research and intervention processes.

Both studies juxtapose seclusion reduction with measures of staff confidence, with both studies finding no discernible difference among staff in their confidence to manage aggression, their perceived safety and their attitude towards seclusion.

### Research with high patient involvement associated with reduction in restrictive practices

A further eight studies were classified as having high patient involvement with patients in the role of partners across all three stages [38, 44, 50, 57, 64, 66, 73, 77]. Four of the eight high involvement studies were conducted in the UK [38, 66, 73, 77], two in Australia [44, 50], one in Canada [57] and one in the Netherlands [64]. Seven of the eight studies explored mechanisms and processes whereby the use of restrictive practices could be reduced. The remaining study explored the reduction of suicide and suicide ideation using technology alongside traditional mental health support measures [44].

The Avon and Wiltshire study [77] used co-production techniques and personal stories to design safety specific interventions and positive behaviour safety plans that included '4 steps to safety' and ‘Safewards’. Lombardo [66] used the PROMISE framework which was co-designed with researchers, trust staff and patients and centred on core values of caring responses to distress and the courage to challenge decisions. Patients were involved in the co-production of the research proposal, research documentation and implementation strategies which sought novel solutions in the delivery of mental health care. Both Lombardo [66] and the CQC report found [73] patient involvement in advisory groups and ward meetings led to a reduction in physical restraints, the former from 328 to 241 to 210 (36%) in consecutive years 2014, 2015/16 and 2016/17 respectively and the latter by 20% across the five participating Trusts. The CQC reported that this was achieved by experts by experience co-delivering staff training and the use of rapid reflection tools post-incident. Lombardo et al. [66] were also able to reduce prone restraints from 82 to 32 (61%) between 2015 and 2017, and the CQC [73] by 70%. Avon and Wiltshire [77] reduced the number of restraints by 97% from 32 to 1 per month between August 2017 and February 2019. Riahi [57] used ‘6 core strategies’ to reduce restraint frequency by 19.7% and the average duration of restraint by 38.9% between April and June 2011.

The ‘6 core strategies’ (cited above) include leadership for cultural change and post-incident debriefing. Service users and families were also involved in care plans and the former were employed in various departments, committee and advisory groups. A Service User Experience Team further communicated service user concerns, compliments and questions to management.

Both Ashcraft [50] and Riley [38] used ‘No Force First’, the former to reduce chemical restraint for those in crisis and the latter to eliminate general restraint. ‘No Force First’ involves executive commitment, ward peer support, risk sharing, and recovery focused, trauma informed care. Ashcraft further informed families and guardians throughout  the entire care process as a means of moving away from compliance oriented care. This was reinforced by the employment of peer support specialists by the unit to create a blended workforce. Ashcraft’s [50] use of ‘No Force First’ led to the range of restraint incidence declining to between 0 and 1.27% of individuals per month, compared to a state-wide incidence of 3.9%. Riley’s [38] use of ‘No Force First’ saw a 27% reduction in assaults on staff with the pilot phase of the study on two wards, reducing physical restraint by over 60%. Between April 2016 and August 2017 Riley’s [38] use of ‘No Force First’ across all inpatient areas reduced restraint by 37% from baseline. Like the CQC [73], Riley et al. [38] fed service user narratives of seclusion and restraint into staff training and education and service users were involved in the initial research project engagement sessions held at each unit. Post study, service users helped to devise a new staff training curriculum which they co-delivered.

Like the highest involvement studies, Fluttert [64] focused on forensic mental health, using the Early Recognition Method (ERM) to reduce aggressive incidents and seclusion by 52.5% from 219 in the Treatment As Usual (TAU) group to 104 in the ERM group. The ERM is a collaboration between nurses and patients to detect the perceptions, thoughts and behaviours that may lead to patient aggression early on. Seclusion rates per person, per month declined from a mean of 0.13 to 0.05, whilst severity declined from 1.38 to 0.50 (as calculated by the Incident Severity Index). Finally, Melvin [44] researched the design and implementation of a co-designed smartphone app to reduce suicide ideation, urges and completions. The app was initially designed with input from individuals with lived experience of suicide ideation and was subsequently modified by service user input in terms of the desirability and functionality of the app’s features and the potential barriers to its future use. Whilst no significant difference in suicide resilience was observed, the severity of suicide ideation reduced from a mean of 4.33 at baseline to 2.29 post intervention (as measured by the Columbia Suicide Severity Rating Scale). There was also a significant increase in the frequency of suicide related coping strategy use between a baseline mean of 22.29 and a post intervention mean of 27.29.

Methodologically, three of the eight high involvement studies were difficult to evaluate for quality as data were not fully reported [66, 73, 77]. Lombardo [66] reported the survey and frequency of restraints data more thoroughly than the qualitative data in an exploration of the relationship between restraint numbers and patient experience. The authors simply note that patient experience surveys (n = 4591) between 2014 and 2017 rated overall satisfaction with care at 87% across the whole period but there is no indication of satisfaction levels before the study commenced. The CQC report [73] aligned the intervention with the study aims and used appropriate statistical analysis whilst Avon and Wiltshire [77] did not report their method fully beyond the use of a series of QI interventions. Ashcraft [50] did  not report complete outcome data and Riahi [57] did not report on the representativeness (or not) of the study sample whereas Fluttert [64] did, reporting a male only sample. The three remaining high involvement studies were of good methodological quality and reported methodological details fully according to MMAT [24].

Of the eight high involvement studies, two used Quantitative Descriptive methods [57, 73], two a series of QI initiatives [38, 77], two Quantitative Non-randomised methods [50, 64], one a principally Qualitative method [66] (though a survey and measures of restraint frequency were reported secondary methods), and one used a Mixed-Methods design [44]. The most common component of interventions in the eight high involvement studies and of their reported success in reducing restrictive practices was collaboration and co-production in the design of policies, procedures and environments with a common subtheme of peer support meetings and patient involvement in ward rounds/meetings.

### Low patient involvement in research associated with more varied focus across studies

Eleven studies were evaluated as having the lowest levels of patient involvement [33, 35, 39, 40, 42, 45, 54, 60, 71, 74, 76]. All eleven studies involved patients as co-thinkers in one stage only. Eight studies involved patients as co-thinkers in the preparation stage only [33, 35, 39, 40, 45, 71, 74, 76], two studies as co-thinkers in the implementation stage only [42, 54], and one as co-thinkers in the execution stage only [60]. Four of the eleven studies focused on restrictive practices [39, 40, 42, 54], two focused on self-harm [71, 74], two on violence [45, 60], and one each on sexual safety [35], general safety [33] and emotional and psychological safety [76]. Restrictive practice interventions in low patient involvement studies included the design of a post seclusion patient information leaflet [39], a seclusion and restraint decision making framework [40], introduction of a sensory modulation room [42] and staff de-escalation training [54]. Self-harm interventions included risk assessment/predictive tools [71] and patient input into the National Suicide Prevention Strategy [74]. Violence interventions included patient preferences input into the DASA [60] and Camberwell Assessment of Needs Forensic Version-Short (CANFOR-S) risk assessment tools [45]. The general safety study [33] emphasised the limits of physical space (ward/building design) as a safety intervention [33], the sexual safety study used qualitative workshops to facilitate sexual safety becoming an ‘always event’ [35], and the emotional/psychological safety study used leadership development and ward rounds as interventions [76].

Whilst the 11 low patient involvement studies included a wide range of safety interventions, over half (n = 6) used a qualitative methodological design and were of good quality but their qualitative design did not allow for measurement of effectiveness. Of the remaining five low patient involvement studies one did not report any intervention outcome whilst four studies with quantitative design reported some intervention effectiveness (i.e. safety improvement). These four studies ranged from methodologically poor [76] through average (Wale) [54] to good (Lloyd [42] and Lantta [60]). Thus, no firm conclusions can be drawn between low patient involvement, methodological quality and intervention effectiveness in improving safety in acute mental health care generally.

### Low patient involvement in research associated with less reduction in restrictive practices

A different picture emerges, however, when a comparative analysis is conducted on those studies focusing specifically on the reduction or elimination of restrictive practices in acute mental health. The two low patient involvement studies that focused on this aspect of safety were less successful, overall, than their high involvement counterparts in reducing restrictive practices [42, 54]. Lloyd [42] succeeded in reducing the rate but not the duration of seclusion episodes, though the former was reduced by 66%, whereas Wale [54] reported reductions in the duration of restraint episodes by 28%, and of seclusions by 27%. However, Wale's reductions in rates of restraint and seclusion failed to reach statistical significance [54]. Lloyd [42] further reported a reduction in the mean core of patient distress as rated by patients pre and post session using a qualitative questionnaire and a 10-point ordinal rating scale, from 6.58 to 3.72, and a reduction in patient injuries by 56%.

Whilst the low patient involvement studies were not necessarily of reduced methodological quality the lowest patient involvement study was evaluated as having the poorest (reported) methodology [35]. Indeed, some low patient involvement studies were methodologically of good quality and had the potential for high patient involvement but the patient voice became a little lost (e.g. in the reporting of results in Curtis [33] only a quarter of quotations are from patients/carers (11/40 and mainly in text)) or patients remained in the background [60]. On the other hand, some lower patient involvement studies were of lower reported methodological quality having, for example, no clear research question or explanation of the research process [39].

### Do studies with high levels of patient involvement and of good methodological quality lead to more effective safety interventions in acute mental health?

The answer to this question is both ‘yes’ and ‘no’. As noted above, the two highest patient involvement studies [37, 43] both reported significant increases in safety in acute mental health settings as a result of their respective interventions, though Qurashi more so than Maguire. Both studies focused on the reduction of restrictive practices in the forensic setting and were evaluated as being of good methodological quality, though again Qurashi slightly more so than Maguire (the latter did not report staff response rate). This might suggest that high patient involvement and good methodological quality do lead to increased safety outcomes in some areas of acute mental health; in this instance safety in the forensic setting. As also noted above, studies with less patient involvement reported intervention effectiveness but at lower levels.

Looking at acute mental health overall, other studies in this review, which were of good methodological quality but had some of the lowest levels of patient involvement [30, 32, 47], also reported improving safety in significant and measurable ways. All focused on non-forensic mental health care and used a range of measures in assessing safety, including the reduction of violent incidents, of seclusion rates and adverse events. Still other studies, with poor (reported) methodological quality *and* the lowest levels of patient involvement, also improved safety [76]. One study that reported an ineffective intervention was assessed as being of good methodological quality and involved patients in the more  active role of partner [36].

From this review it is therefore impossible to draw any firm conclusions about the relationship between methodological quality, patient involvement and increased safety across the field of acute mental health. This could be the subject of a future study where outcome measures, patient involvement, context and methodology are standardised. Of note, however, is the large number of grey literature sources that were assessed as being of poor methodological quality. This may well be a reporting issue and, if this is the case, suggests that a template enabling the standardised reporting of research methods and patient involvement in safety across both published and grey literature sources may benefit the field. Finally, 14 of the 52 studies reviewed here did not report an intervention outcome at all, making the drawing of insightful conclusions even more difficult [50, 53, 62, 65, 67–69, 72, 74, 75, 77, 78, 80, 81].

## Discussion

To the authors’ knowledge, this review is the first of its kind to explore the extent of patient involvement in researching the design, development and implementation of patient safety interventions in acute mental healthcare. The findings suggest that studies are diverse in terms of patients’ roles in developing safety interventions, and the stage and extent to which patients are involved. Overall, research reporting higher levels of patient involvement tended to focus on restrictive practices, involving patients as either co-thinkers, advisors or partners, whereby patients were asked for their opinion, provided unsolicited advice or worked in equal partnership with researchers and clinicians [21]. The ongoing research focus on restrictive practices may be symptomatic of a prevailing risk averse culture [32, 33, 80] that potentially impedes the development of therapeutic environments [82–84]. To a much lesser extent, patients were involved as decision makers and were thus less able to use their initiative in driving decisions around safety interventions, in part perhaps because of ongoing power imbalances and the paternalistic characteristics of clinical settings [85, 86]. Patients were also most commonly involved in a single stage of the research process, as opposed to being involved throughout an intervention’s design, development and implementation, which could be interpreted as tokenistic involvement [21].

Our finding that patients continue to be involved in more passive research roles in safety research in acute mental health is a finding noted by others in health research generally [87] and the reasons for this are multi-faceted. Patients can feel insecure about taking on more active roles and tasks in research [88]. In the U.K. in particular a scepticism prevails regarding many kinds of community engagement and this can result in tokenistic public involvement [89, 90]. Further barriers to patient involvement include time and compensation for patients [87], and for researchers, funding, logistics, recruitment, researchers’ own skills to involve patients and adequate institutional support in doing so [87].

Importantly, almost all interventions reported here showed some improvement to the safety of care, qualitatively and/or quantitatively, supporting earlier theoretical approaches to patient safety (e.g. [68, 91–96]. However, the significance of such improvements varied and specific detail surrounding how improvements were achieved was often minimal. Nevertheless, the findings presented here support Weich et al. [97] who reject the stance that active service users cannot effectively contribute to patient safety in mental health settings. This review shows that they can and do, even in the arguably more challenging forensic mental health context.

The majority of the studies reporting high levels of patient involvement focused on restrictive practices and evidenced improvements in patient safety, some of which were maintained longer term. Additionally, five of the eight high involvement studies involved patients in the co-production of policy, procedures and environmental design as their primary intervention and ward rounds or meetings as their secondary intervention, highlighting areas of opportunity for organisations to learn and implement effective involvement. Specifically, the two studies with the highest levels of patient involvement [37, 43] both showed safety improvements compared to their lower patient involvement counterparts [42, 54]. However, these two studies [37, 43] researched seclusion and restraint in the forensic mental health, which is arguably where patient involvement may be more pivotal to success.

Overall, no direct association was found between high patient involvement, good methodological quality and improved safety. That is to say, some studies with good methodological quality but low patient involvement still reported intervention effectiveness (Lloyd). However, no study with high patient involvement reported an ineffective intervention, regardless of methodological quality.

The finding that studies with lower levels of patient involvement evidenced some improvements in safety does not necessarily indicate patient involvement is not necessary in safety research. The underpinning rationale for patient involvement according to Martin et al. [99] suggests that even in the absence of technocratic improvements to safety, the involvement of patients may serve an important moral and ethical purpose in enabling those who use services to actively contribute to the design and delivery of them, which has been more recently supported (e.g. [100, 101]). Indeed, studies with less patient involvement showed more diversity in terms of their safety focus, including relatively novel approaches to self-harm, violence, sexual safety and psychological safety interventions. These studies engaged patients in the role of co-thinker in one stage only, primarily in the preparation stage, with interventions ranging from personalised collaborative risk assessments to environmental design. Such, studies may be considered at the forefront of research, as is often the case with qualitative research design. Whilst patient involvement in these studies was  low, this  will perhaps lead progressively to increased patient involvement as part of larger, future studies. However, risks of low patient involvement include frontloading expenditure of resources towards involvement activity, whilst patient views are disregarded or hold relatively little weight in comparison to other stakeholders over time. This is a longstanding issue in safety interventions across settings and raises potential ethical issues demonstrated in well documented reports (e.g. [102, 103]). To help to tackle this, involvement of patients in a mental healthcare needs to be more clearly defined, and staff require practical support and guidance to develop the skills and teamwork to facilitate patient involvement, as well as develop trusting relationships with patients and mindfulness about how this work fits with the wider organisational culture [97, 104].

Overall, the Involvement Matrix [21] offered a valuable lens through which to view the included studies. But an important omission of Smit’s Involvement Matrix [21] is that it does not account for the extent to which patient involvement impacted on studies or was valued by researchers and staff [105]. Additionally, many studies reviewed here did not describe in detail the nature of patient involvement, lending its inclusion, particularly in study titles and abstracts, a tokenistic quality. Further, many studies did not use the term ‘involvement’ or did not report patient and staff data with parity [33]. Other studies promised more patient involvement than appeared to be delivered in practice [39]. These issues make thorough evaluation difficult, and may reflect authorship or publishing priorities and support the argument for use of a standardised patient involvement reporting template, of which Smit et al.’s [21] Involvement Matrix is one example. Smit et al.’s [21] Involvement Matrix did, however, prove useful in indicating areas under development, weakness and, perhaps most importantly, omissions, enabling analysis of patient involvement in research and interventions beyond the descriptive level.

In summary, findings from this review suggest that patient involvement in research and interventions to improve safety in acute mental health should be actively encouraged at policy level as safety improvements are reported. However, patient involvement should not be tokenistic in terms of either patient roles or stages of involvement. The initial engagement of patients in research and interventions should also translate into improvements that are meaningful for patients, who have a right to be involved. While it might be expected that patient involvement in mental healthcare safety improvement is synonymous with the nature of the discipline underpinned by a history of user activism, this review aligns with previous research in suggesting that it is one of the most challenging areas across the health service in which to achieve this, as involvement is poorly understood, engaged with to varying extents and patient satisfaction remains relatively low [72, 73, 82, 97, 106, 107]. As this review has shown, while high levels of patient involvement may play an important role in improving patient safety, it alone may be insufficient to improve safety in acute mental healthcare. We need a better understanding of how patients perceive safety and of how their involvement in designing interventions makes these more impactful, potentially identifying key involvement points for patients. Moreover, the principal focus on restrictive practices across all studies reported here suggests that physical safety continues to be the primary concern in mental health safety. In the UK context, this stands alongside concerns over use of the Mental Health Act [108], which is currently under review. [12] Future research may address both concerns.

### Limitations

Despite an inclusive search strategy, relevant articles may not have been identified if they were not indexed to the databases searched. Additionally, poor reporting or missing data within the included studies may have led to an unduly negative assessment of quality. Some studies report only one stage of an intervention [63] with other stages awaiting publication as an intervention was  rolled out. Some inconsistency between researchers’ evaluation of studies in relation to MMAT and Smits’ intervention matrix [21] was a possibility, though sample cross checking between researchers was conducted to minimise this. Similarly, the classification of studies by topic and involvement type were open to alternate interpretations. For example, studies classified by intervention type under ‘tool use/development’ may also (or alternately) have been classified under ‘culture change’ [46] or roles could be interpreted as involving listening or co-thinking (e.g. patient involvement in debrief techniques). Furthermore, studies in this review were evaluated to be of high involvement only if patients were involved in all three stages, even if they involved patients as partners rather than decision makers. Conversely, studies were evaluated as low involvement if patients were involved in one stage only and in the less engaged roles of listener or co-thinker. Others may choose to evaluate such studies differently, demonstrating the subjective element of the Involvement Matrix.

## Conclusion

There is evidence that patient safety can be improved in acute mental healthcare settings when patients are involved in interventions. However, a tendency for involvement to focus on restrictive practices and involve patients in limited ways and only in certain stages of research is apparent, suggesting that there may still be a way to go for many organisations to culturally embrace patient involvement as a valued method of meaningfully improving safety in acute mental health settings. This  review makes a valuable contribution to the field, with direct relevance and utility for a wide range of stakeholders including policy makers, service providers, commissioners, healthcare staff and patients, presenting both challenges to, and help for, attending to the importance of patient involvement in patient safety interventions in acute mental healthcare.

## Data Availability

Not applicable.

## Appendix 1 Summary of the Involvement Matrix (Adapted from Smits et al. [21] Reproduced with permission of the Centre of Excellence for Rehabilitation Medicine, Utrecht)

**Table:**

PPI	Definition	Application in this review
Role		Type of involvement
Listener	Given information	Patient given information on admission to a facility
Co-thinker	Is asked for opinion	Involvement in ward meetings Provides information
Advisor	Gives (un)solicited advice	Unsolicited *opinions* given in the context of data collection Co-developing Co-production
Partner	Works as an equal partner	Delivery of patient stories Worked together to make decisions about study and consulted throughout Co-produce and use the tool to self-manage Co-delivering training Support worker role
Decision-maker	Takes initiatives and/or makes decisions	Consumer consultants on project management groups

Stage		Extent of involvement
Preparation	Planning	All pre project activities excluding research/intervention design. May include recruitment of service users and/or third sector participants, funding and ethics applications, input into preparation of materials e.g. interview schedules, participant information sheets, questionnaires. All pilot activities
Execution	Design	All/any aspects of research project/intervention methodological design- deciding what is going to be done-how answering the research question or assessing the intervention is executed
Implementation	Putting into practice	Participation in any aspect of the conduct of the research or rolling out of the intervention, e.g. conducting interviews, distributing questionnaires, participating in the conduct of focus groups. Steering group membership. May involve dissemination activities

## Appendix 2: Search terms used for database search

1. Population
  (Patient* or client or "service user" or consumer or carer or relative or inpatient or family).mp. [mp=title, abstract, heading word, table of contents, key concepts, original title, tests & measures, mesh].
2. Intervention
  (patient* or client or "service user" or consumer or carer or relative or inpatient or family) ADJ2 (safety or "safety incident" or "adverse event" or harm or "preventable harm" or "safety intervention" or "never event" or "serious incident" or "serious incidents requiring investigation" or "near miss" or "medical error" or "prescribing error" or "close call" or "service quality incident" or "process of care problem" or "undesirable event" or "medication misadventure" or risk or "shared-decision making" or "shared decision making" or "co-design" or "participatory action research" or "experience based co-design" or "experience based design" or "quality improvement" or intervention or involvement or contribution or "challenge staff" or "question staff" or "help staff" or "speak up" or "speak up campaign" or satisfaction or perception or feedback or opinion or experience or complain* or report or concern or voice or concern)
3. Outcome
  (violence or "self-harm" or "self harm" or "self-injury" or "self injury" or suicide* or restraint or seclusion or tranquilisation or "health related quality of life" or "negative event" or "treatment compliance" or "treatment concordance" or readmission or "preventable harm" or "ward atmosphere" or "well-being" or "well being" or "safety incident" or "safety outcome" or "routinely collected data" or "safety data" or "incident report" or "monitoring safety" or "safety reporting" or "physical safety" or "psychological safety" or "patient safety outcome" or morbidity or "health status" or "incident rate" or "safety measure" or "adverse event" or harm or "never event" or "serious incident" or "serious incidents requiring investigation" or "near miss" or "medical error" or "prescribing error" or "close call" or "undesirable event" or "unsafe care experience" or "adverse drug event" or "service quality incident" or "quality of care" or "quality of life" or "quality of healthcare" or "quality indicator" or "quality assurance" or "quality outcome" or "symptom outcome" or "error" or "under diagnosis" or "over diagnosis" or "failure of diagnosis" or "misdiagnosis" or "safety culture" or "safety climate" or abscond or "escorted leave" or "unescorted leave" or "racial attack" or "sexual attack" or "physical attack" or "verbal attack" or "missing patient" or "self discharge" or "failure to return from authorised leave" or "qualitative" or "quantitative" or "focus group" or interview or "quality improvement project" or "ethnography" or "observation").mp. [mp=title, abstract, heading word, table of contents, key concepts, original title, tests & measures, mesh]
4. Setting
  (("mental health" or "mental illness" or psycho* or schizo* or depress* or "mental disorder" or psychiat* or "psychiatric illness" or "psychiatric disorder" or suicide or "suicide attempt" or suicidal or "self harm" or "self-harm" or "self-injury" or "self injury" or anxiety or "mood disorder" or bipolar or psychotic or "chronic mental health" or "severe mental health" or "mentally ill" or paranoid or paranoia) adj2 (hospital* or NHS or Trust or ward or inpatient or acute or "secondary care" or unit or facility or centre or department or service)).mp. [mp=title, abstract, heading word, table of contents, key concepts, original title, tests & measures, mesh]

## Abbreviations

AI: Artificial intelligence
CAG: Consumer Advisory Group
CANFOR-S: Camberwell assessment of needs forensic version-short
CQC: Care Quality Commission
DASA: Dynamic appraisal of situational aggression
ERM: Early recognition method
MeSH: Medical subject headings
MMAT: Mixed methods appraisal tool
NFF: No force first
NICE: National Institute for Health and Care Excellence
NRLS: National Reporting and Learning System
PDSA: Plan, Do, Study, Act
PICOS: Population, intervention, comparison, outcomes, study design
PICU: Psychiatric Intensive Care Unit
PRISMA: Preferred reporting items for systematic reviews in meta-analyses
PROMISE: PROactive management of integrated services and environments
QI: Quality improvement
TAU: Treatment as usual
UK: United Kingdom
US: United States
WHO: World Health Organsiation

## Footnotes

1 The number of deaths exceeds the number of safety incidents classified as ‘severe’ and thus the former number must include safety incidents classified as of moderate and/or low severity.

2 ‘6 core strategies’ are: supportive leadership; continual workforce development; consumer involvement; use of seclusion reduction assessment and planning tools, use of data; debriefing.
