# Correction: To what extent are patients involved in researching safety in acute mental healthcare?

**DOI:** 10.1186/s40900-023-00469-8

**Published:** 2023-08-09

**Authors:** Lyn Brierley-Jones, Lauren Ramsey, Krysia Canvin, Sarah Kendal, John Baker

**Affiliations:** 1 School of Healthcare, University of Leeds, Leeds, UK; 2 Yorkshire Quality and Safety Research Group, Bradford Institute for Health Research, Bradford, UK; 3 Leeds Institute of Health Sciences, Leeds, UK

**Correction: Research Involvement and Engagement (2022) 8:8** 10.1186/s40900-022-00337-x

Following publication of the original article [1], the authors reported errors in Appendix 3: Prisma flow chart and would like to correct the number of studies under the heading **Search strategy and study selection**. The revised Appendix 3: Prisma flow chart and the Search strategy and study selection section is indicated hereafter and the changes in the given section have been highlighted in **bold typeface**.

The incorrect Search strategy and study selection section reads:

After full-text screening, a further **248** papers were excluded leaving a total of 23 studies from the published literature search.

The correct Search strategy and study selection section should read:

After full-text screening, a further **249** papers were excluded leaving a total of 23 studies from the published literature search.

Appendix 3: Prisma flow chart should read:

**Appendix 3: Prisma flow chart**


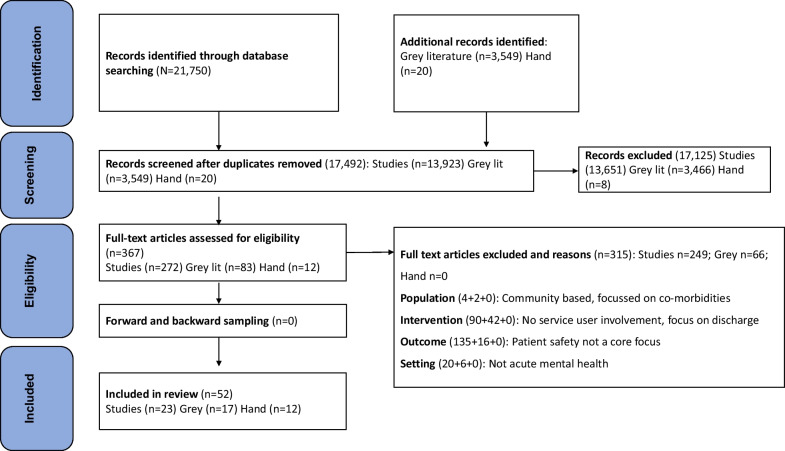
All the changes requested are implemented in this correction and the original article [1] has been corrected.
